# Association of frailty and chemotherapy-related adverse outcomes in geriatric patients with cancer: a pilot observational study in Taiwan

**DOI:** 10.18632/aging.203673

**Published:** 2021-11-08

**Authors:** Ya-Wen Ho, Woung-Ru Tang, Shih-Ying Chen, Shu-Hui Lee, Jen-Shi Chen, Yu-Shin Hung, Wen-Chi Chou

**Affiliations:** 1 Division of Hematology and Oncology, Department of Internal Medicine, Chang Gung Memorial Hospital at Linkou and College of Medicine, Chang Gung University, Taoyuan, Taiwan; 2 School of Nursing, College of Medicine, Chang Gung University, Taoyuan, Taiwan; 3 Department of Nursing, Linkou Chang Gung Memorial Hospital, Chang Gung University of Science and Technology, Cardinal Tien Junior College of Healthcare and Management, Taoyuan, Taiwan

**Keywords:** chemotherapy-related adverse outcomes, comprehensive geriatric assessment, frailty, geriatric patients with cancer

## Abstract

Background: With the rapid growth of the elderly population and the increasing incidence of cancer, an increasing number of geriatric patients are receiving cancer treatment, making the selection of appropriate treatment an important issue. Increasing studies have confirmed that frailty can predict adverse outcomes in geriatric patients with cancer after treatment, but local data from Taiwan are lacking. Therefore, this study aimed to investigate the correlation between frailty and chemotherapy-related adverse outcomes in geriatric patients with cancer.

Material and Methods: A total of 234 geriatric patients aged ≥65 years with cancer receiving chemotherapy were enrolled during the study period of September 2016 to November 2018. The collected data included: patients’ basic demographics and Comprehensive Geriatric Assessment (CGA) before treatment, chemotherapy-related adverse outcomes, unexpected hospitalizations, and emergency department visits within 3 months of treatment. We investigated the association between frailty and chemotherapy-related adverse outcomes in geriatric patients with cancer using the chi-square test and logistic regression analysis.

Results: The prevalence of frailty in geriatric patients with cancer was 58.1%. Age, marital status, main caregiver, cancer type, and Eastern Cooperative Oncology Group performance status, and physical fitness were factors associated with frailty. Frail geriatric patients with cancer were at higher risk of chemotherapy-related adverse outcomes, such as grades 3–4 thrombocytopenia (odds ratio [OR] = 11.13, *p* = 0.021) and grades 3–4 hyponatremia (OR = 12.03, *p* = 0.017), than non-frail patients, and they were at increased risk of unexpected hospitalizations (OR = 2.15, *p* = 0.025) and emergency department visits (OR = 1.99, *p* = 0.039).

Conclusions: Frailty is a common problem in geriatric patients with cancer and significantly impacts chemotherapy-related adverse outcomes. Our findings suggest that geriatric patients with cancer should undergo frail assessment prior to chemotherapy as a reference to guide future treatment decisions.

## INTRODUCTION

When the proportion of the population aged 65 years or older reaches 7% of the total population internationally, an “aging society” is created; when it reaches 21%, a “super-aged society” is created [1]. Taiwan officially became an aging society in March 2018 and is expected to become a super-aged society in 2026 due to the dual effects of a declining birth rate and increased aging [1], which shows that the population is changing at a rapid pace. Therefore, the issue of geriatric health care is urgent and important.

Cancer has ranked first among the top 10 causes of death in Taiwan for 38 years in a row [2]. Aging is a high risk factor for cancer [3, 4]. According to the World Health Organization and the International Agency for Research on Cancer, approximately 7 million people aged 65 or older were newly diagnosed with cancer in 2012, and the number of geriatric patients with cancer is expected to reach 14 million by 2035 [4]. Moreover, as many as 70% of all cancer-related deaths occur in patients aged 65 or older [5]. This is also the case in Taiwan, where the rate of cancer deaths in elderly individuals aged 65 or older is increasing annually [6]. This indicates that the future care demand for geriatric patients with cancer will pose enormous social and economic challenges.

Cancer treatment for the elderly is complex and requires multiple considerations. Multiple chronic diseases, polypharmacy, and decreased physical fitness due to physiological aging may affect cancer diagnosis and treatment or even increase the risk of treatment [7–9]. However, despite the high incidence of newly-diagnosed cancer in patients over 65 years of age (51.6%) [10], geriatric patients are excluded from most oncology studies because of healthcare provider bias, patient age, comorbidity, and poor physical fitness [11, 12]. The lack of guidelines for the treatment of geriatric patients with cancer due to their underrepresentation in clinical trials and the paucity of available empirical data [12] has led to limited anticancer treatment options for geriatric patients with cancer.

Frailty is a popular topic in geriatrics in recent years. Frailty is defined as a decrease in the reserve capacity of the body’s multiple systems that increases the risk of adverse health outcomes because the body’s adaptability and resilience cannot respond when stressful events occur [13–18]. Frailty can be used to predict chemotherapy-related adverse outcomes such as mortality [19, 20], chemotherapy drug tolerance [16, 21], severe toxicity [20, 22], treatment interruption, and hospitalization [23] in geriatric patients with cancer undergoing chemotherapy. Therefore, the American Society of Clinical Oncology [24], National Comprehensive Cancer Network [25], and International Society of Geriatric Oncology [26] state that the Comprehensive Geriatric Assessment (CGA) should be used to assess the frailty of geriatric patients before they receive cancer treatment. The above guidelines, however, are recommendations from Western countries, whereas research on frailty in cancer patients in Taiwan remains in its infancy. Only a few studies have evaluated the association between frailty and chemotherapy-related adverse events in geriatric patients with cancer in Asia [27]. Due to the lack of literature on frailty in local cancer patients, this study aimed to investigate the correlation between frailty and chemotherapy-related adverse outcomes in geriatric patients with cancer in Taiwan for use as a basis for clinical healthcare decisions to improve the safety of treatment of geriatric patients with cancer.

## RESULTS

### Patients’ basic attributes

A total of 234 geriatric patients with cancer were enrolled in this study (median age, 70 years; range, 65–96 years). The majority of patients were female (53%), were married (80.8%), had solid organ tumors (67.5%), had stage III cancer (40.2%), and received polypharmacy (82.9%). Advanced age, non-married status, non-spouse as the main caregiver, lymphoma, and poor ECOG performance status were significantly associated with patient frailty (*p* < 0.05) (Table 1).

**Table 1:** Correlation between basic attributes and frailty of geriatric patients with cancer.

**Variable**	**Overall (*N* = 234)** ***n* (%)**	**Non-frail (*N* = 98)** ***n* (%)**	**Frail (*N* = 136)** ***n* (%)**	***P***
Age, Median (range)	70 (65–96)	68 (65–85)	72 (65–96)	**0.007**
65–69	103 (44.0)	56 (57.1)	47 (34.6)	
70–74	67 (28.6)	23 (23.5)	44 (32.4)	
75–79	45 (19.2)	14 (14.3)	31 (22.8)	
≥80	19 (8.2)	5 (5)	14 (10.3)	
Gender				0.99
Female	124 (53.0)	52 (53.1)	72 (52.9)	
Male	110 (47.0)	46 (46.9)	64 (47.1)	
Marriage				**0.014**
Married	189 (80.8)	87 (88.8)	102 (75.0)	
Others	45 (19.2)	11 (11.2)	34 (25.0)	
Education				0.74
Junior high school or less	145 (62)	59 (60.2)	86 (63.2)	
Senior high school or more	89 (38)	39 (39.8)	50 (36.8)	
Occupation				0.85
No	203 (86.8)	86 (87.8)	117 (86.0)	
Yes	31 (13.2)	12 (12.2)	19 (14.0)	
Main caregiver				**0.047**
Spouse	121 (51.7)	60 (61.2)	61 (44.9)	
Child	87 (37.2)	29 (29.6)	58 (42.6)	
Others	26 (11.1)	9 (9.2)	17 (12.5)	
Smoking				0.99
No	155 (66.2)	65 (66.3)	90 (66.2)	
Yes	79 (33.8)	33 (36.7)	46 (33.8)	
Drinking				0.45
No	165 (70.5)	66 (67.3)	99 (72.8)	
Yes	69 (29.5)	32 (32.7)	37 (27.2)	
ECOG performance				**<0.001**
0	136 (58.1)	79 (80.6)	57 (41.9)	
1	86 (36.8)	19 (19.4)	67 (49.3)	
≥2	12 (5.2)	0 (0.0)	12 (8.8)	
Cancer type				<0.001^*^
Hematological cancer	76 (32.5)	19 (19.4)	57 (41.9)	<0.001^#^
Solid cancer	158 (67.5)	79 (80.6)	79 (58.1)	0.006^#^
Breast	52 (22.2)	31 (31.6)	21 (15.4)	
Colorectal	46 (19.7)	22 (22.4)	24 (17.6)	
Lung	12 (5.1)	5 (5.1)	7 (5.1)	
Stomach	11 (4.7)	5 (5.1)	6 (4.4)	
Urogenital	10 (4.3)	3 (3.1)	7 (5.1)	
Others	27 (11.5)	13 (13.3)	14 (10.3)	
Stage				0.07
I	20 (8.5)	7 (7.1)	13 (9.6)	
II	80 (34.2)	39 (39.8)	41 (30.1)	
III	94 (40.2)	42 (42.9)	52 (38.2)	
IV	40 (17.1)	10 (10.2)	30 (22.1)	
Chemotherapy regime				0.99^*^
Monotherapy	40 (17.1)	17 (17.3)	23 (16.9)	0.81^#^
5-Fluorouracil or capecitabine	26 (11.1)	13 (13.3)	13 (9.6)	
Cisplatin	9 (3.8)	4 (4.1)	5 (3.7)	
Gemcitabine	5 (2.1)	0	5 (3.7)	
Combination therapy	194 (82.9)	81 (82.7)	113 (83.1)	0.99^#^
R-CHOP	76 (32.8)	19 (19.4)	57 (41.9)	
XELOX or FOLFOX	41 (17.5)	19 (19.4)	20 (14.7)	
CEF	26 (11.1)	16 (16.3)	8 (5.9)	
CMF	25 (10.7)	13 (13.3)	12 (8.8)	
GC	19 (8.1)	8 (8.1)	11 (8.1)	
Others	7 (3.0)	6 (6.1)	1 (0.7)	

Abbreviations: ECOG: Eastern Cooperative Oncology Group; R-CHOP: rituximab plus cyclophosphamide plus doxorubicin plus vincristine plus prednisone; XELOX: capecitabine plus oxaliplatin; FOLFOX: oxaliplatin plus 5-fluorouracil and leucovorin; CEF: cyclophosphamide plus epirubicin plus 5-fluorouracil; CMF: cyclophosphamide plus methotrexate plus 5-fluorouracil; GC: gemcitabine plus cisplatin. ^*^p value for two groups. ^#^*p* value for subgroups.

### Frailty prevalence and common deficient dimensions

The prevalence of frailty was 58.1% among the 234 patients. On the CGA, nutrition (65.4%) was the most deficient dimension, followed by comorbidity (38.5%), functional status (24.8%), and polypharmacy (23.1%). Mood (17.5%), falls (13.2%), cognition (10.7%), and social support (9.4%) were less often deficient (Table 2).

**Table 2:** Deficient dimensions of the comprehensive geriatric assessment (*N* = 234).

**Frailty dimension**	**Measure**	**Number of items**	**Score range**	**Cutoff value**	***N* (%)**
Functional status	ADL	10	0–100	<100	58 (24.8)
	IADL	8	0–8	<8	
Cognition	Modified_short version MMSE	13	0–13	<9	25 (10.7)
Nutrition	MNA-SF	6	0–14	<12	153 (65.4)
Mood	GDS-4	4	0–4	>1	41 (17.5)
Social support	Living alone or lack of family support	1	Yes/No	Yes	22 (9.4)
Polypharmacy	Number of medications	1	0-∞	>4	54 (23.1)
Comorbidity	CCI	19	0-33	>1	90 (38.5)
Mobility/Falls	Number of falls	1	0-∞	>1	31 (13.2)

Abbreviations: ADL: activities of daily living; CCI: Charlson comorbidity index; GDS: geriatric depression scale; IADL: instrumental activities of daily living; MMSE: mini mental state exam; MNA-SF: mini nutritional assessment-short form.

### Analysis of frailty and chemotherapy-related adverse outcomes

The analysis of frailty by level of toxicity showed that among all grades of toxicities, frailty was significantly associated with low hemoglobin (74.5% vs 92.6%, *p* < 0.001), hypokalemia (8.2% vs 21.3%, *p* = 0.006), infection (17.3% vs 32.4%, *p* = 0.01), and neuropathy (16.3% vs 35.3%, *p* = 0.002). In the classification of grade 3–4 toxicity, frailty was significantly associated with thrombocytopenia (1.0% vs 10.3%, *p* = 0.005), any non-hematological toxicity (25.5% vs 39.0%, *p* = 0.035), and hyponatremia (1.0% vs 11.0%, *p* = 0.003) (Table 3).

**Table 3:** Correlation between frailty and chemotherapy-related adverse events (*N* = 234).

**Adverse events**	**Non-frail (*n* = 98)**		**Frail (*n* = 136)**		***P* for all grades**	***P* for grade 3–4**
	**All grades, *n* (%)**	**Grade 3–4, *n* (%)**	**All grades, *n* (%)**	**Grade 3–4, *n* (%)**		
Any hematological toxicity	89 (90.8)	34 (34.7)	131 (96.3)	54 (39.7)	0.097	0.495
Low hemoglobin	73 (74.5)	7 (7.1)	126 (92.6)	20 (14.7)	**<0.001**	0.097
Thrombocytopenia	47 (48.0)	1 (1.0)	69 (50.7)	14 (10.3)	0.69	**0.005**
Leukopenia	50 (51.0)	16 (16.3)	72 (52.9)	29 (21.3)	0.79	0.40
Neutropenia	59 (60.2)	29 (29.6)	76 (55.9)	45 (33.1)	0.59	0.67
Neutropenic fever	5 (5.1)	5 (5.1)	15 (11.0)	15 (11.0)	0.15	0.15
Any non-hematological toxicity	94 (95.9)	25 (25.5)	133 (97.8)	53 (39.0)	0.46	**0.035**
Excessive AST/ALT	40 (40.8)	1 (1.0)	51 (37.5)	4 (2.9)	0.68	0.40
Excessive creatinine	19 (19.4)	0	39 (28.7)	4 (2.9)	0.13	0.14
Hyponatremia	8 (8.2)	1 (1.0)	24 (17.6)	15 (11.0)	0.05	**0.003**
Hypokalemia	8 (8.2)	4 (4.1)	29 (21.3)	10 (7.4)	**0.006**	0.405
Hyperglycemia	30 (30.6)	4 (4.1)	55 (40.4)	12 (8.8)	0.13	0.195
Oral mucositis	21 (21.4)	1 (1.0)	43 (31.6)	3 (2.2)	0.10	0.64
Infection	17 (17.3)	10 (10.2)	44 (32.4)	26 (19.1)	**0.01**	0.068
Hypertension	83 (84.7)	14 (14.3)	112 (82.4)	19 (14)	0.72	0.99
Nausea/vomiting	42 (42.9)	0	53 (39)	3 (2.2)	0.59	0.27
Fatigue	41 (41.8)	1 (1.0)	74 (54.4)	1 (0.7)	0.06	0.99
Diarrhea	21 (21.4)	1 (1.0)	31 (22.8)	3 (2.2)	0.87	0.64
Neuropathy	16 (16.3)	0	48 (35.3)	0	**0.002**	−

For chemotherapy-related adverse outcomes, grade 1–2 toxicity occurred in 20% or more of patients, or grade 3–4 toxicity occurs in 5% or more of patients before being shown in the table above. ^a^Neutropenia in CTCAE has no cases of grade 1–2 toxicity. Abbreviations: AST: aspartate aminotransferase; ALT: alanine aminotransferase.

The logistic regression analysis suggested that frail geriatric patients with cancer were at higher risk of grade 3–4 thrombocytopenia (odds ratio [OR] = 11.1; 95% confidence interval [CI], 1.44–86.14; *p* = 0.021) and grade 3–4 hyponatremia (OR = 12.0; 95% CI, 1.56–92.64; *p* = 0.017) than those without frailty (Table 4).

**Table 4:** Frailty and risk of chemotherapy-related adverse outcomes.

**Variable**	**Group**	**OR (95% CI)**	***P***	AOR^*^	***P***	**AOR^#^**	***P***
Grade 3–4 thrombocytopenia	Non-frail	1 (reference)		1		1	
	Frail	11.1 (1.44–86.1)	0.021	10.2 (1.26–90.1)	0.031	8.9 (1.17–92.4)	0.040
Grade 3–4 hyponatremia	Non-frail	1 (reference)		1		1	
	Frail	12.0 (1.56–92.6)	0.017	11.6 (1.44–94.1)	0.024	9.9 (1.40–99.1)	0.036
Unexpected hospitalizations	Non-frail	1 (reference)		1		1	
	Frail	2.15 (1.10–4.17)	0.025	2.09 (1.06–5.22)	0.032	1.87 (1.03–6.29)	0.043
Emergency department visits	Non-frail	1 (reference)		1		1	
	Frail	1.99 (1.03–3.82)	0.039	1.72 (1.01–3.22)	0.045	1.49 (1.00–4.21)	0.048

Abbreviations: CI: confidence interval; OR: odds ratio; AOR: Adjusted odds ratio. ^*^Adjusted for age and gender. ^#^Adjusted for age, gender, cancer types, and chemotherapy regimens.

**Figure 1:**
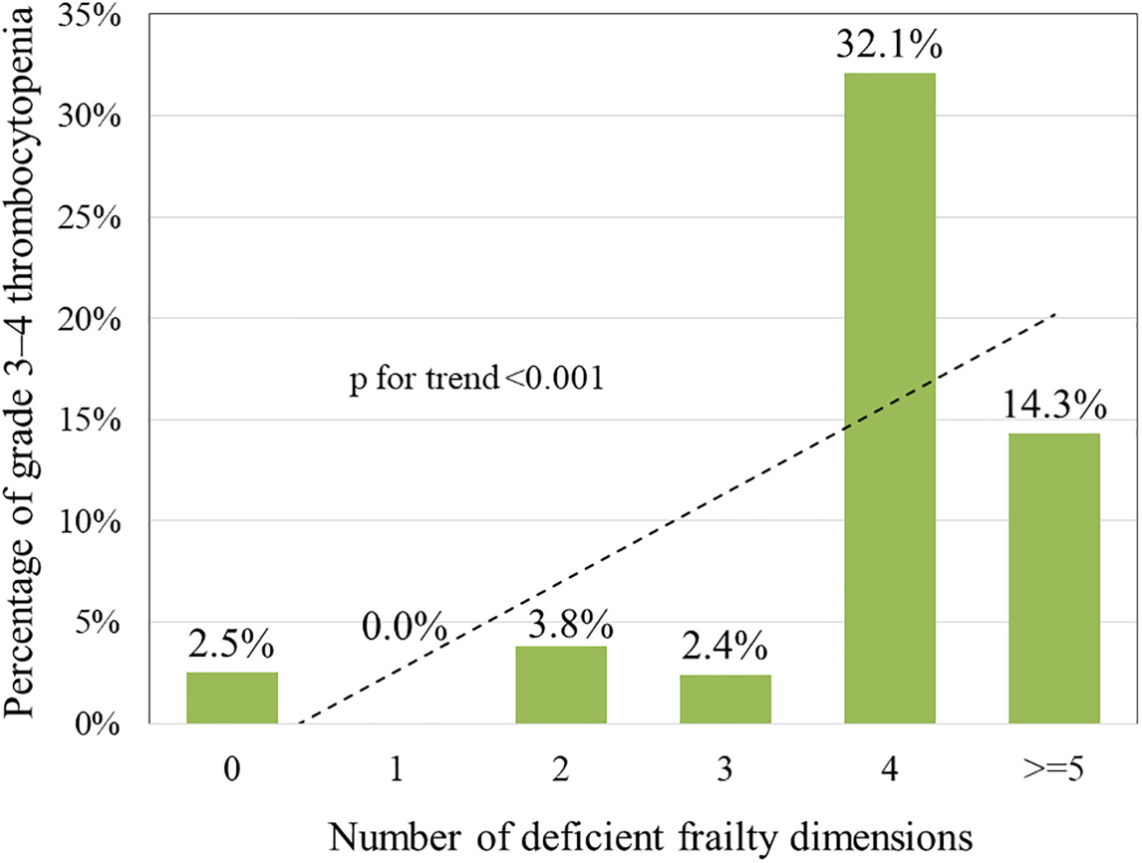
Correlation between number of deficient frailty dimensions and grades 3–4 thrombocytopenia.

**Figure 2:**
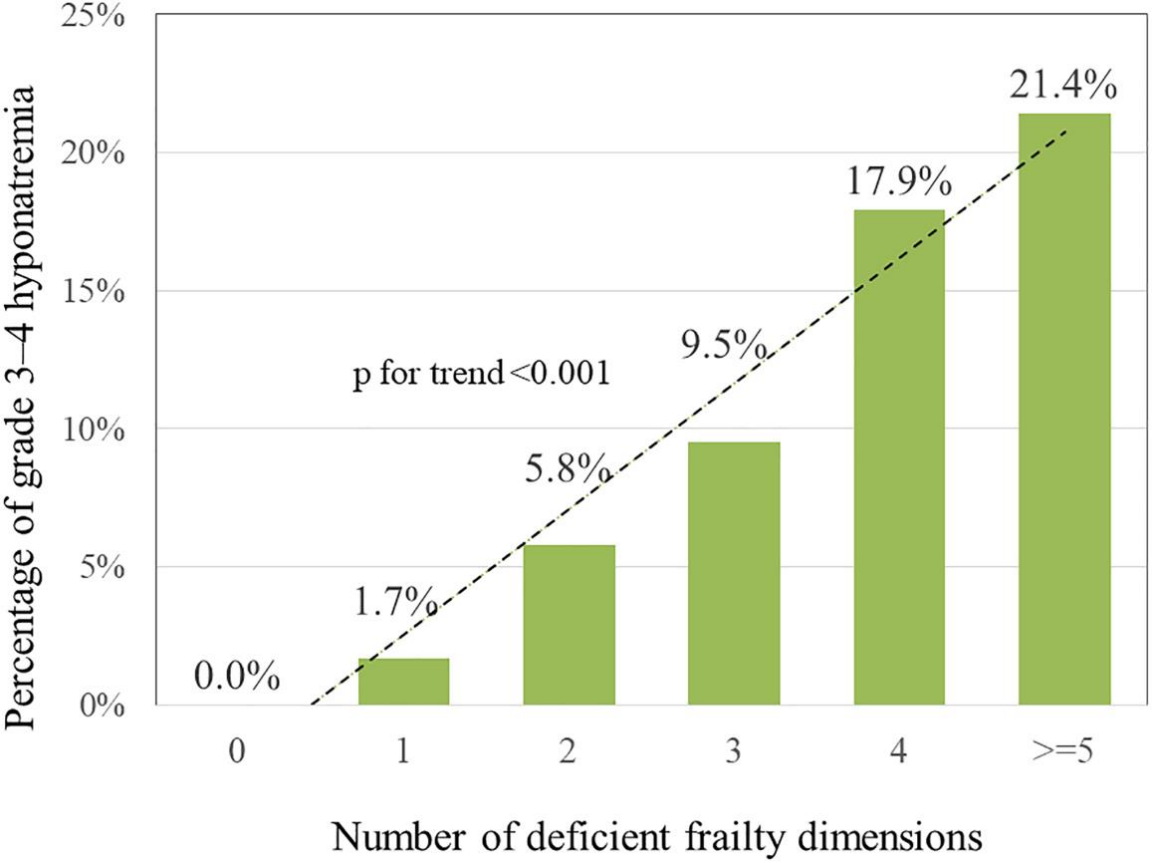
Correlation between number of deficient frailty dimensions and grade 3–4 hyponatremia.

In terms of the number of deficient frailty dimensions, when the number was 0, 1, 2, 3, 4, and ≥5, the proportions of grade 3–4 thrombocytopenia were 2.5%, 0%, 3.8%, 2.4%, 32.1%, and 14.3%, respectively (*p* for trend < 0.001) (Figure 1); the proportions of grade 3–4 hyponatremia were 0%, 1.7%, 5.8%, 9.5, 17.9%, and 21.4%, respectively (*p* for trend < 0.001) (Figure 2). This showed that the incidence of grade 3–4 thrombocytopenia and hyponatremia increased significantly with the number of deficient frailty dimensions.

### Analysis of frailty, unexpected hospitalizations, and emergency department visits

The prevalence of unexpected hospitalizations and emergency department visits within 3 months of treatment was 22.6% and 23.1%, respectively. The incidence of unexpected hospitalizations was 15.3% in the non-frail group and 27.9% in the frail group, which indicated that frail patients were at higher risk of unexpected hospitalizations than non-frail patients (OR = 2.15; 95% CI, 1.10–4.17; *p* = 0.025) (Table 4). For patients with hospitalization, the median length of hospital stay was 8 days (range, 1–26) and 14 days (range, 1–45) for fit and frail patients (*p* = 0.058), respectively. The incidence of emergency department visits was 16.3% and 27.9% in the non-frail and frail groups, respectively, suggesting that frail patients were at higher risk of emergency department visits than non- frail patients (OR = 1.99; 95% CI, 1.03–3.82; *p* = 0.039). The adjusted ORs among each adverse event remain had significant in-groups difference after adjusting for age, gender, cancer types, and chemotherapy regimens.

## DISCUSSION

The present study aimed to examine the association between frailty and chemotherapy-related adverse outcomes among geriatric patients with cancer in Taiwan. Frailty was a common problem in geriatric patients with cancer at a prevalence of 58%. In addition, the risk of chemotherapy-related adverse outcomes, including chemotherapy toxicity, unexpected hospitalizations, and emergency department visits, was significantly higher among frail geriatric patients with cancer when undergoing curative high-intensity chemotherapy than non-frail geriatric patients with cancer. This study is the first of its kind in Taiwan to examine the prevalence of frailty and association of frailty and chemotherapy-related adverse outcomes in geriatric patients with cancer. Our findings suggest that frailty assessments should be performed before geriatric patients with cancer receive chemotherapy. This assists with the early detection of high-risk patients prone to chemotherapy-related adverse outcomes, which not only provides early psychological preparation for patients and their families, it helps healthcare professionals intervene early to prevent the occurrence of adverse outcomes.

An estimated 58% of our geriatric patients with cancer suffered from frailty. A retrospective systemic review showed a 43% median prevalence (range, 7–68%) of frailty in geriatric patients with cancer [21], significantly lower than that in the present study. Of the 16 papers reviewed in this study that used CGA for frailty identification, most assessed CGA in 6–7 dimensions and defined frailty as ≥2 deficient dimensions [21]. The present study used the same cut-off criteria, but its inclusion of more (eight) dimensions in the CGA assessment may have contributed to the higher prevalence of frailty than reported by previous studies.

Age, marital status, main caregiver, and cancer type were correlated with frailty in the present study. Age is the most widely known correlate of frailty, and the individual physical function, psychological function, and social support inevitably deteriorate with aging [28], making frailty assessments and timely interventions paramount for geriatric patients. The high proportion of frail patients who are single and whose main caregiver is not their spouse indicates that family support, especially care from a spouse, is crucial for geriatric patients with cancer. This study showed that geriatric patients with hematological cancers had significantly higher rates of frailty than those with solid tumors (75% vs 50%). We further analyzed that 45% of patients with hematological cancers were ≥75 years old (compared with 19% of patients with solid tumors), while 42% had stage IV cancer (compared with 5% of patients with solid tumors), which may account for the difference in frailty rates. Moreover, the differences in staging and treatment between hematological cancers and solid tumors were so dramatic that more studies are needed to confirm the results of this study and demonstrate whether patients with hematologic cancers are at higher risk of frailty.

Our results showed that poor ECOG performance status was also a risk factor for frailty. Performance is now commonly used in clinical practice to assess the physical fitness of cancer patients as a reference for cancer treatment [29]. Although the ECOG assessment is easy to use, it is too simplified and easily influenced by assessor subjectivity [30, 31]. In this study, patients with an ECOG performance status ≥2 accounted for only 5.2%, but those with physical impairment (activities of daily living [ADL] or instrumental ADL [IADL] deficiency) reached 24.8%, indicating that ECOG may not be an appropriate indicator for physical fitness assessments in geriatric patients. In addition to using ECOG as a simple physical fitness assessment for cancer patients, ADL or IADL should be performed as a physical fitness assessment for geriatric patients with cancer in the future to better predict their frailty and chemotherapy risk.

Similar to previous literature, frailty had a significant impact on the occurrence of grade 3 or above serious adverse outcomes in our cohort [23, 32–35]. The association of grade 3 or above toxicity with frailty has been studied in the literature, but less severe chemotherapy toxicity is also important for geriatric patients with cancer. Grade 1–2 toxicity (hypokalemia and neuropathy) was also associated with frailty in our study. Therefore, for geriatric patients with frailty, the development of less severe toxicity should also be considered, and the treatment regimen should be adjusted promptly to avoid increasing the subsequent damage of the side effects of anticancer treatment, thus improving drug safety and patient quality of life. Contrastingly, some studies indicated that frailty was not associated with the side effects of chemotherapy [36–38]. This may be due to the fact that the study was a pilot trial conducted in older newly-diagnosed cancer patients receiving different antitumor strategies and its findings may not be applicable to geriatric cancer patients underwent chemotherapy [38], the low intensity of chemotherapy received by the study participants [36], and the small number of participants but comparison between multiple groups of frailty, all of which may diminish the number of cases in each group and the failure to achieve significance in the statistical analysis [37].

In the analysis of frailty, unexpected hospitalizations, and emergency department visits, this study revealed that the incidence of unexpected hospitalizations and emergency department visits was higher among frail patients than among non-frail patients, which was concurred with the findings of previous studies [23, 32, 38]. The cumulative effect of negative factors such as older age, poorer physical fitness, and weak family support systems led to higher incidence and severity of CTCAE in frail patients, which resulted in an increased incidence of unexpected hospitalizations and emergency department visits. In this way, appropriate interventions should be provided to improve the frailty of elderly and frail patients before versus after anticancer treatment, and close attention should be paid to the assessment and management of the side effects of chemotherapy to reduce the incidence of serious adverse outcomes.

In published literature, frailty is commonly quantified by counting the number of deficits, including disability, disease, psychosocial distress, and cognitive impairments [16, 24]. The Frailty index (FI), ranging from 0 to 1, is calculated by dividing the number of deficits by the total numbers of variables measured [17]. The index has the advantage because it is constructed from different clinical variables that are relevant to the specific clinical settings, and because the outcome measure has continuous values [39]. However, since the optimal cut-off value of FI might vary among different outcome measures, the clinical application for cancer patients undergoing specific treatment may be limited to specific settings. In the current study, we successfully applied an eight-dimension CGA to identify frail patients who are at the highest risk of adverse outcomes during the chemotherapy course, suggesting that CGA is a useful assessment tool for frailty in Taiwanese geriatric cancer patients. However, there is no consensus on the cutoff level of the numbers and aspects of geriatric domain impairment for CGA in Asian cancer patients. The optimal instruments of CGA suitable for the Asian oncogeriatric population are needed for further exploration and validation.

There are some limitations in this study, including the large number of cancer types enrolled in this study, the wide variation in chemotherapy treatments for different cancer types, and the limitation of the enrollment site to a medical center in northern Taiwan. The provision of healthcare may vary across countries, regions, and hospitals, which can limit the inference of the results. Although the p for trend had a significant association between grade 3–4 thrombocytopenia and numbers of deficient frailty dimensions in our study. These two parameters did not present a linear association in our study. The possible explanation might be because of the small numbers of the event, accounting for only 15 of 234 patients (6.4%) had grade 3–4 thrombocytopenia, in our study. Similarly, the wide confidence interval of odds ratio in risk of chemotherapy-related adverse outcomes indicated either the rare incidence of the events or small numbers of patients, therefore, more patient numbers are necessary to recruit to validate our data. The chemotherapy toxicity of patients in this study was determined by the treating physicians of different departments based on the CTCAE, which is more subjective for judging the adverse outcomes of non-tested values; thus, the incidence and severity of these non-tested values may have been underestimated. Future studies may incorporate the Patient Reported Outcomes-CTCAE) developed by the NCI [40] to eliminate the subjective bias in the definition of side effects by different healthcare professionals.

In conclusion, this study is one of the first studies in Taiwan to investigate the effect of frailty on chemotherapy-related adverse outcomes in geriatric patients with cancer. It revealed a significant effect of frailty on chemotherapy-related adverse outcomes in geriatric patients with cancer. Our findings suggest that frail assessments be conducted before chemotherapy is administered to geriatric patients with cancer in the future. This information will give clinical staff more references when discussing treatment decisions with patients, thus enhancing the quality of care for geriatric patients with cancer in Taiwan.

## MATERIALS AND METHODS

### Participants and data collection

This study was part of a large-scale prospective, longitudinal, and observational study in which data were collected through a structured questionnaire. The study site was a medical center in northern Taiwan. Inclusion criteria were: (1) age ≥65 years and ready to receive chemotherapy with curative intent; (2) have a clear mind and the ability to communicate verbally or in writing; and (3) willingness to sign the respondent’s consent form. The study was approved by the Institutional Review Board (no. 201600916B0). The basic attributes of the participants in the week before treatment were collected, including sex, age, marital status, education, occupation, main caregiver, cancer type, cancer stage, Eastern Cooperative Oncology Group (ECOG) performance status, chemotherapy regimen, smoking history, and drinking history. Data on chemotherapy-related adverse outcomes, unexpected hospitalizations, and emergency department visits within 3 months of treatment were also collected for analysis.

### Comprehensive geriatric assessment

All patients were assessed with the Comprehensive Geriatric Assessment (CGA) in person by two research assistants with medical management and nursing backgrounds based on a daily working within one week before initiation of chemotherapy. The CGA assessment dimensions in this study included: functional status [41, 42], cognition [43], nutrition [44], mood [45], social support [46], polypharmacy [25], comorbidity [47], and falls [24]. Based on our previous validation study using the same assessment tool in a Taiwanese adult cancer population, frailty in this study was defined as the presence of two or more frail conditions [48]. The assessment tools and cut-off points for each dimension are presented in Table 2.

### Chemotherapy-related adverse outcomes, unexpected hospitalizations, and emergency department visits

In this study, chemotherapy-related adverse outcomes were evaluated within 3 months of treatment according to the Common Terminology Criteria for Adverse Events (CTCAE) version 4.0 [49] published by the National Cancer Institute (NCI). The CTCAE has five levels of severity, with a higher level indicating more severe side effects (toxicity) experienced by the patient. The study also collected data on whether patients had unexpected hospitalizations and emergency department visits within 3 months of treatment.

### Statistical analysis

We adopted the chi-square test to analyze the correlation between basic patient attributes and frailty as well as the correlation between frailty and chemotherapy-related adverse outcomes. The variables showing significant correlations between frailty and chemotherapy-related adverse outcomes were then subjected to binary logistic regression analysis to understand the association between frailty and chemotherapy-related adverse outcomes, unexpected hospitalizations (yes/no), and emergency department visits (yes/no).

Chemotherapy-related adverse outcomes were analyzed in three different classifications: all grades (with/without toxicity), grades 1–2 (with/without grade 1–2 toxicity), and grades 3–4 (with/without grade 1–2 toxicity) to examine the performance of frailty according to different grades of toxicity. The data were analyzed using the SPSS 20.0 for Windows statistical software package, and all statistical analyses were two-tailed, with values of *p* < 0.05 considered statistical significance.

### Ethics approval and consent to participate

This study was approved by the institutional review board of Chang Gung Memorial Hospital in August 2017 (ethic code: 1608080002) and has been conducted in compliance with the Helsinki Declaration (1996).

### Availability of supporting data

The datasets used and/or analyzed during the current study are available from the corresponding author on reasonable request.
